# Minimally invasive esophagectomy for cancer in COVID hospitals and oncological hubs: are the outcomes different?

**DOI:** 10.1007/s10353-022-00751-1

**Published:** 2022-03-18

**Authors:** Pamela Milito, Emanuele Asti, Marco Resta, Luigi Bonavina

**Affiliations:** 1 Department of Biomedical Sciences for Health, IRCCS Policlinico San Donato, Division of General and Foregut Surgery, University of Milan, Piazza Malan 1, 20097 San Donato Milanese (Milano), Italy; 2 Department of Anesthesiology, IRCCS Policlinico San Donato, San Donato Milanese (Milano), Italy

**Keywords:** Esophageal carcinoma, Laparoscopy, Thoracoscopy, SARS-CoV-2 infection, Respiratory complications

## Abstract

**Introduction:**

The outbreak of coronavirus disease 2019 (COVID-19) has caused significant delays in oncological care worldwide due to restriction of elective surgery and intensive care unit capacity. It has been hypothesized that COVID-free oncological hubs can provide safer elective cancer surgery compared to COVID hospitals. The primary aim of the present study was to analyze the outcomes of minimally invasive esophagectomy for cancer performed in both hospital settings by the same surgical staff.

**Methods:**

All esophagectomies for cancer performed during the pandemic by a single team were reviewed and data were compared with control patients operated during the preceding year. Screening for severe acute respiratory syndrome coronavirus type 2 (SARS-CoV-2) was performed prior to surgery, and special precautions were taken to mitigate hospital-related transmission of COVID-19 among patients and healthcare workers.

**Results:**

Compared to the prepandemic period, the esophagectomy volume decreased by 64%. Comorbidities, time from onset of symptoms to first visit, waiting time between diagnosis and surgery, operative approach and technique, and the pathological staging were similar. None of the patients tested positive for COVID-19 during in-hospital stay, and esophagectomy was associated with similar outcomes compared to control patients.

**Conclusion:**

Outcomes of minimally invasive esophagectomy for cancer performed in a COVID hospital after implementation of a COVID-free surgical pathway did not differ from those obtained in an oncological hub by the same surgical team.

## Main novel aspects

Implementation of a COVID-free surgical pathway may guarantee optimal outcomes of minimally invasive esophagectomy for cancer.

## Introduction

The coronavirus disease 2019 (COVID-19) outbreak has disrupted the pattern of healthcare and caused significant delays in oncological care worldwide due to restriction of elective surgery and intensive care unit capacity. Major surgical procedures were concentrated in hospitals with COVID-free surgical pathways or in oncological centers receiving patients from COVID hospitals. Northern Italy was particularly hard hit by the COVID-19 outbreak, with more than 130,000 deaths during the first two waves of the disease (spring 2020 and autumn/winter 2020/2021). During the first wave of the pandemic, Policlinico San Donato hospital was converted to a COVID-19 facility, and the activity of the Esophageal Cancer Center was subjected to restrictions depending on the availability of intensive care beds. Later on, the Regional Health Council converted cancer-specialized hospitals into oncological hubs to guarantee safer and effective surgical care during the pandemic. The surgical team of Policlinico San Donato was then granted access to the facilities of the European Institute of Oncology which served as an oncological hub. However, the hypothesis that oncological hubs can provide safer elective cancer surgery compared to well-organized COVID hospitals remains to be proven [1]. The primary aim of the present study was to analyze the outcomes of minimally invasive esophagectomy (MIE) for cancer performed in both hospital settings by the same surgical staff.

## Patients and methods

The study was approved by the Internal Review Board (PSD 077, December 1, 2021) and was conducted in accordance with the principles set out in the Helsinki declaration. All data of patients diagnosed with esophageal cancer and referred to our tertiary care Esophageal Cancer Center were prospectively collected using a dedicated Institutional database. A retrospective analysis using an anonymized dataset was conducted to compare the outcomes of minimally invasive esophagectomy (MIE) performed in two different hospital settings during the first and second wave of COVID-19 (from March 7, 2020 through March 31, 2021) by the same surgical staff. Consecutive patients with esophageal cancer were included in this study. Collected data comprised demographics, smoking habits, previous history of cancer, hypertension, diabetes, cardiovascular disease, chronic obstructive pulmonary disease, histological type, disease stage, previous chemo/radiation therapy, type of surgical procedure, pathological results, length of hospital stay, immediate postoperative complications, and short-term clinical outcomes. These variables were compared with data from control patients treated during the year before the outbreak.

### Perioperative management and surgical technique

**Fig. 1:**
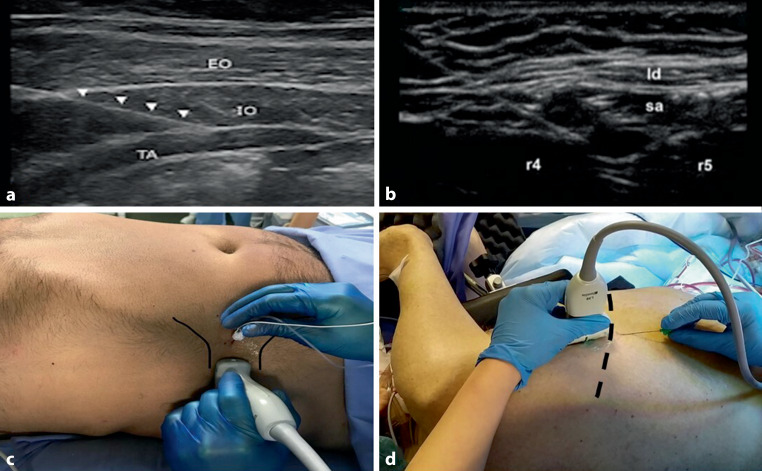
Pre-emptive locoregional anesthesia using ultrasound-guided transversus abdominal plane (TAP) block (**a–c**), and serratus anterior plane (SAP) block (**d**). *EO* External oblique, *IO* internal oblique, *TA* Transversus Abdominis

**Fig. 2:**
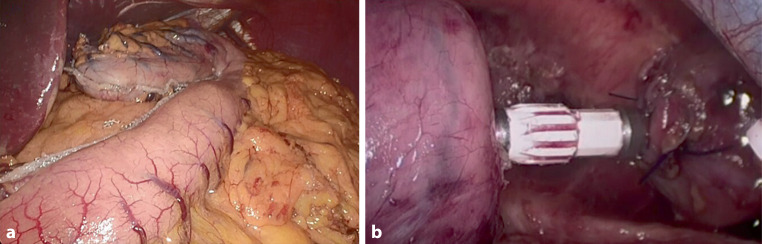
Laparoscopic and thoracoscopic Ivor Lewis esophagectomy. **a** Stapled gastric tubulization. **b** Esophagogastric anastomosis with circular stapler

**Fig. 3:**
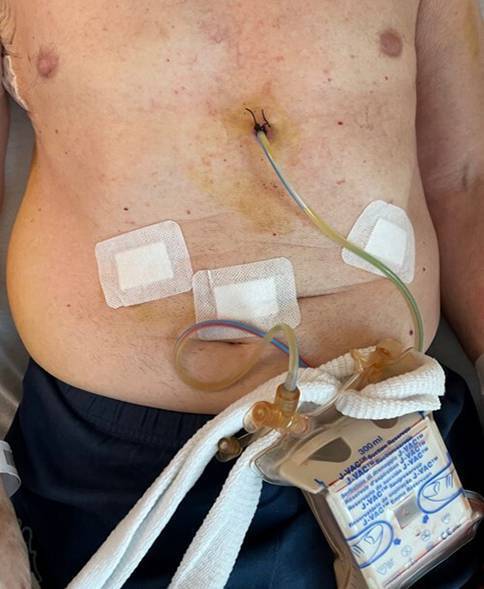
Trans-hiatal mediastinal drainage with portable reservoir (J-VAC; J&J, New Brunswick, NJ, USA) as a strategy to avoid intercostal pain and minimize use of opioids

**Fig. 4:**
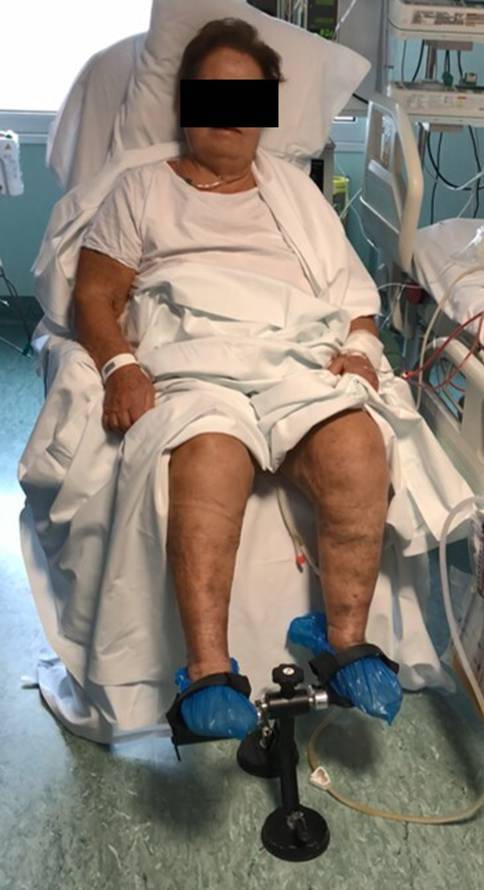
The protocol of enhanced recovery after surgery includes early physical exercise and ambulation

**Fig. 5:**
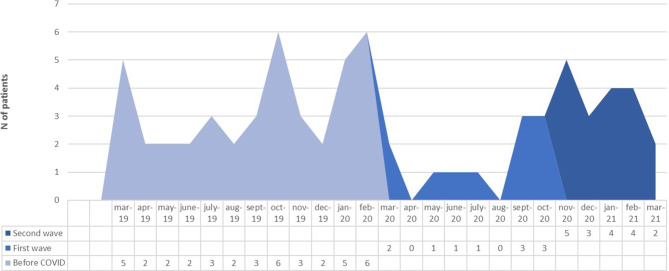
Over time distribution of esophagectomies before and during the pandemic period

Screening for severe acute respiratory syndrome coronavirus type 2 (SARS-CoV-2) started 2 weeks prior to surgery. A negative nasopharyngeal swab with a polymerase chain reaction (PCR) test and a negative chest X‑ray/computed tomography (CT) chest scan were required at the time of prehospitalization. A COVID-19-free surgical pathway was implemented. All procedures that could generate aerosol particles (upper gastrointestinal endoscopy, bronchoscopy, endotracheal intubation/extubation, chest tube insertion, laparoscopy and thoracoscopy) were performed by healthcare personnel with the highest level of personal protection equipment [2]. Before surgery, transversus abdominis plane (TAP) and serratus anterior plane (SAP) block were performed by the anesthesiology team (Fig. 1). The surgical procedures consisted of a two-stage hybrid Ivor Lewis (laparoscopy and right thoracotomy) or a three-stage McKeown (thoracoscopy, laparoscopy, and left cervicotomy) esophagectomy with gastric conduit reconstruction ([3]; Fig. 2). Patients were transferred to a semi-intensive ward or to the intensive care unit (ICU) for the first night after surgery. Pain was managed with ropivacaine 5% through a perifascial thoracic catheter, and intravenous paracetamol or ketorolac was administered as needed [4]. Traditional intercostal drainage was replaced by trans-hiatal drain through the subxyphoid trocar site (Fig. 3; [5, 6]). A standardized protocol for enhanced recovery was routinely applied (Fig. 4). Use of spirometer incentive was discontinued during the pandemic. In the absence of fever and with negative pleural drain amylase sampling and normal serum C‑reactive protein on postoperative day 2 and 3, respectively, an early semiliquid diet was permitted [7]. A gastrografin swallow study was generally performed on postoperative day 4. Visitors were not allowed, and communication with patients was only through videocalls and social media.

### Statistical analysis

Continuous data are summarized as median and interquartile range (IQR). Categorical variables are shown as frequencies and percentage. Categorical variables were compared using Χ^2^ or Fischer’s exact test as appropriate, and Mann–Whitney *U *test was used for continuous variables. A *p* value less than 0.05 was considered statistically significant.

## Results

Fig. 5 shows the monthly distribution of esophagectomies during the study period. Overall, there was a 64% reduction of esophageal cancer surgery volume compared to the prepandemic period. None of the patients tested positive for SARS-CoV‑2 either in the preoperative or postoperative course. There were no significant differences regarding preoperative patients’ characteristics and perioperative outcomes (Table 1).

**Table 1:** Comparison of demographic and clinical characteristics of patients undergoing esophagectomy before and during the COVID-19 pandemic

	2019 *n* = 41	COVID hospital (First wave, *n* = 11)	Oncological hub (Second wave, *n* = 18)	*p*
*Sex, M/F*	29/11	9/2	13/5	0.813
*Age, years, median (IQR)*	63.0 (13.8)	63.0 (14.8)	65.0 (14.0)	0.432
*BMI, kg/m*^*2*^*, median (IQR)*	23.8 (5.4)	24.8 (4.1)	24.1 (4.4)	0.991
*Smokers*	20 (48.8)	6 (54.5)	5 (27.8)	0.572
*Comorbidities, n (%)*				
Diabetes	7 (17.0)	2 (18.2)	6 (33.3)	0.329
Hypertension	14 (34.1)	3 (27.3)	12 (66.7)	0.031
Dyslipidemia	8 (19.5)	2 (18.2)	6 (33.3)	0.307
Cardiovascular disease	2 (4.8)	1 (5.6)	1 (5.6)	0.999
*Days from onset of symptoms to first visit, median (IQR)*	88.0 (119.0)	44.0 (66.0)	73.0 (112.3)	0.156
*Histology, n (%)*				0.142
Adenocarcinoma	28 (68.3)	6 (54.5)	14 (77.8)	
Squamous-cell carcinoma	9 (21.9)	2 (18.2)	4 (22.2)	
Other	4 (9.8)	3 (27.3)	0 (0.0)	
*Tumor location, n (%)*				0.106
Upper third	1 (2.4)	0 (0.0)	0 (0.0)	
Medium third	12 (29.2)	4 (36.4)	4 (22.2)	
Lower third	22 (53.7)	2 (18.2)	7 (38.9)	
Esophagogastric junction	6 (14.6)	5 (45.5)	7 (38.9)	
*Days from first visit to admission, median (IQR)*	147.5 (106.5)	97.0 (213.8)	135.5 (138.8)	0.955
*NACT/NACRT, n (%)*	24 (58.5)	4 (36.4)	13 (72.2)	0.235
*Number of CT cycles, median (IQR)*	3.0 (4.0)	4.5 (1.8)	4.0 (1.0)	0.281
*Surgical approach, n (%)*				0.106
Hybrid Ivor Lewis	26 (63.4)	8 (72.7)	17 (94.4)	
Totally mini-invasive Ivor Lewis	7 (17.1)	0 (0.0)	0 (0.0)	
McKeown thoracolaparoscopic	6 (14.6)	3 (27.3)	1 (5.6)	
Esophagocoloplasty	2 (4.9)	0 (0.0)	0 (0.0)	
*Conversion to open, n (%)*	0.0 (0)	0 (0.0)	0 (0.0)	NA
*Number of retrieved nodes, median (IQR)*	26.0 (15.0)	33.0 (22.5)	25.5 (14.0)	0.530
*T Stage, n (%)*				0.411
pT0	5 (12.2)	4 (36.3)	2 (11.1)	
pT1	10 (24.4)	1 (9.0)	2 (11.1)	
pT2	8 (19.5)	0 (0.0)	3 (16.7)	
pT3	15 (36.6)	6 (54.5)	10 (55.6)	
pT4	3 (7.3)	0 (0.0)	1 (5.6)	
*N stage, n (%)*				0.890
pN0	17 (41.4)	6 (54.5)	8 (44.4)	
pN+	24 (58.5)	5 (45.5)	10 (55.6)	
*Clavien–Dindo grade, n (%)*				0.726
Grade II	3 (7.3)	1 (9.0)	1 (5.6)	
Grade IIIA	3 (7.3)	2 (18.0)	2 (11.1)	
Grade IIIB	6 (14.6)	0 (0.0)	1 (5.6)	
Grade IV	1 (2.4)	0 (0.0)	1 (5.6)	
*Length of stay, median (IQR)*	10.0 (4.0)	11.5 (8.8)	20.0 (16.0)	0.119
*30-day mortality, n (%)*	2 (4.8)	0 (0.0)	0 (0.0)	0.482

*BMI* Body mass index, *CT* chemotherapy, *IQR* Interquartile Range, *NACT* neo-adjuvant chemotherapy, *NART* neo-adjuvant radiotherapy

## Discussion

This study shows that MIE for cancer during the pandemic period was not associated with higher morbidity and mortality, and that outcomes did not differ among patients undergoing surgery in the COVID hospital and in the oncological hub. Despite the initial concerns raised by SAGES and EAES regarding the possible spread of infection through surgical smoke during laparoscopic procedures [8], we did not change our routine surgical approach. Of note, there was no instance of conversion to open surgery, and the rate and grade of complications were similar compared to control patients.

With none of the patients developing SARS-CoV‑2, the respiratory complications were directly related to surgery and were comparable with the prepandemic period. It has previously been shown that surgery is safe in patients who are appropriately screened for SARS-CoV‑2 preoperatively, and that the risk of 30-day mortality is associated with age >70 years, American Society Anesthesiologists (ASA) score 3–5, cancer surgery, and major surgery [9]. Also, dedicated COVID-19-free surgical pathways have been shown to provide safe elective cancer surgery. Dolan et al. recommend prioritization of surgical care for patients with esophageal cancer. They reported that esophagectomy can be safely performed, with appropriate precautions, also in major and well organized COVID hospitals with dedicated intensive care unit and inpatient wards [10]. We recognize that the results of the present study may not be generalizable because of the limited number of patients, selection bias due to possible shift toward nonsurgical therapy, and other measurable and unmeasurable factors in the two different hospital settings.

## Conclusions

Implementation of a COVID-free surgical pathway can guarantee optimal outcomes of minimally invasive esophagectomy for cancer.
